# Supplementary material for: Increasing incidence of reported scabies infestations in the Netherlands, 2011–2021
Source: PLoS One. 2022 Jun 24;17(6):e0268865. doi: 10.1371/journal.pone.0268865 (PMC9231777; doi:10.1371/journal.pone.0268865)
Supplement: S1 Database — (DOCX) [file pone.0268865.s001.docx]

|  | **Incidence** |
| --- | --- |
| **Year** | **per 1.000** |
| 2011 | 0.6 |
| 2012 | 0.6 |
| 2013 | 0.9 |
| 2014 | 1.0 |
| 2015 | 1.2 |
| 2016 | 1.7 |
| 2017 | 1.9 |
| 2018 | 2.1 |
| 2019 | 2.1 |
| 2020 | 2.6 |

**Source: Nivel Primary Care Database**

| **Scabies incidence per 1.000** |  |  |  |  |  |  |  |  |  |  |  |  |  |  |
| --- | --- | --- | --- | --- | --- | --- | --- | --- | --- | --- | --- | --- | --- | --- |
| **Year** | **0-4 year** | **5-9 year** | **10-14 year** | **15-19 year** | **20-24 year** | **25-29 year** | **30-34 year** | **35-39 year** | **40-44 year** | **45-49 year** | **50-54 year** | **55-59 year** | **60-64 year** | **65+ year** |
| 2011 | 0.49 | 0.49 | 0.32 | 0.88 | 1.33 | 1.09 | 0.50 | 0.33 | 0.45 | 0.66 | 0.70 | 0.54 | 0.46 | 0.25 |
| 2012 | 0.39 | 0.44 | 0.50 | 0.93 | 1.67 | 1.53 | 0.83 | 0.63 | 0.51 | 0.48 | 0.46 | 0.44 | 0.40 | 0.23 |
| 2013 | 0.71 | 0.74 | 0.66 | 1.93 | 2.73 | 1.76 | 0.98 | 0.95 | 0.61 | 0.72 | 0.81 | 0.74 | 0.42 | 0.31 |
| 2014 | 0.79 | 0.79 | 1.17 | 1.68 | 3.15 | 1.61 | 1.06 | 0.95 | 0.88 | 0.90 | 0.84 | 0.79 | 0.46 | 0.30 |
| 2015 | 0.96 | 1.01 | 1.08 | 2.16 | 4.22 | 2.29 | 1.10 | 0.90 | 1.08 | 1.00 | 1.14 | 0.93 | 0.63 | 0.40 |
| 2016 | 1.44 | 1.38 | 1.54 | 3.45 | 5.88 | 2.70 | 1.34 | 1.39 | 1.32 | 1.23 | 1.58 | 1.00 | 0.79 | 0.82 |
| 2017 | 1.64 | 1.86 | 1.83 | 4.39 | 6.75 | 3.42 | 1.78 | 1.54 | 1.30 | 1.62 | 1.63 | 1.23 | 0.83 | 0.45 |
| 2018 | 1.39 | 2.13 | 1.95 | 3.82 | 6.87 | 3.50 | 2.06 | 1.75 | 1.82 | 1.97 | 1.87 | 1.41 | 1.09 | 0.60 |
| 2019 | 2.07 | 2.46 | 2.06 | 4.14 | 6.77 | 3.52 | 2.26 | 1.53 | 1.68 | 2.07 | 1.63 | 1.72 | 1.25 | 0.57 |
| 2020 | 2.51 | 2.49 | 2.61 | 4.51 | 9.19 | 4.48 | 2.64 | 2.23 | 1.95 | 2.33 | 2.46 | 2.10 | 1.27 | 0.59 |
